# Acoustic Enhancement Performance of Hierarchical ZSM-5 Zeolites with Different Si/Al Ratios

**DOI:** 10.3390/nano15110797

**Published:** 2025-05-26

**Authors:** Mingbo Guo, Yijun Wang, Lei Zhang, Junran Lu, Chang Gong, Wanning Zhang, Yuxi Fang, Xinyuan Zhu, Shunai Che

**Affiliations:** 1 State Key Laboratory of Synergistic Chem-Bio Synthesis, School of Chemical Science and Engineering, Frontiers Science Center for Transformative Molecules, Shanghai Key Laboratory for Molecular Engineering of Chiral Drugs, Shanghai Jiao Tong University, 800 Dongchuan Road, Shanghai 200240, China; mbguo_jeff@sjtu.edu.cn (M.G.); zhangwanning@sjtu.edu.cn (W.Z.); sjtu15901600323@sjtu.edu.cn (Y.F.); 2 SSI New Material (Zhenjiang) Co., Ltd., 7 Songlin Mountain Road, Zhenjiang 212006, China; yj.wang@n-bass.com (Y.W.); steven.zhang@n-bass.com (L.Z.); jr.lu@n-bass.com (J.L.); chang.gong@n-bass.com (C.G.)

**Keywords:** ZSM-5, acoustic enhancement materials, hierarchical structure, mesoporous zeolite, microspeaker

## Abstract

Size restrictions pose increasing challenges to the acoustic performance of microspeakers in portable devices as the size of such devices, and thus the back volume of microspeakers, continues to shrink. Filling the back volume with porous materials, such as zeolites, has been proved to be an effective strategy for improving acoustic performance. In this work, hierarchically structured ZSM-5 zeolites with abundant mesopores were synthesized via the traditional hydrothermal method by adjusting the SiO_2_/Al_2_O_3_ ratios (SAR), and their pore structures and morphologies were systematically investigated. Their acoustic enhancement performance was evaluated using a commercial microspeaker. Based on their acoustic properties, the influence of pore structure on acoustic performance was further studied. The ZSM-5 zeolite sample with an SAR of 614, which exhibited the maximum mesopore volume, demonstrated exceptional acoustic enhancement performance with a resonance offset of 199.53 Hz and an enhanced sound pressure level of 4.74 dB at 500 Hz. The presence of mesopores significantly facilitates diffusion within the zeolite crystals, enabling air molecules to access more micropores for efficient sorption–desorption processes during diaphragm vibration in microspeakers. Furthermore, supermicropores were found to contribute to improved performance by adsorbing air molecules during diaphragm vibration, complementing the role of micropores.

## 1. Introduction

The improvement of the acoustic performance of microspeakers in mobile phones has become a major challenge as the size of microspeakers continues to shrink [1]. Filling the back volume of microspeakers with porous materials, referred to as “acoustic enhancement materials” (AEMs), has been recognized as an effective approach and has achieved remarkable success [2]. However, research on AEMs remains limited, and they are often mistakenly categorized as “sound-absorbing materials” (SAMs). This misunderstanding has hindered progress in AEMs, leaving their underlying mechanisms unclear.

Microspeakers are transducers that convert electrical signals into sound energy and are widely used in cell phones [3], laptops [4], and other portable electronic devices. A typical loudspeaker system consists of a speaker unit and an enclosure, which is usually sealed to prevent interference from the sound waves generated by the speaker unit. This sealed enclosure, commonly referred to as the “back volume”, typically occupies approximately 40% of the total volume of a microspeaker in a mobile phone [5]. The acoustic performance of microspeakers is typically evaluated based on resonance frequency (*f*_0_) and sound pressure level (SPL), with lower *f*_0_ and higher SPL generally indicating better acoustic performance [6]. Shiah et al. [7] demonstrated that the size of the back volume significantly impacts the low-frequency performance (100~1000 Hz) of loudspeakers, and increasing the back volume can enhance acoustic performance. However, the demand for high-quality acoustic performance in mobile devices poses a challenge due to the trend toward smaller, slimmer, and lighter microspeakers in modern device design. As a result, the available space for the back volume is reduced, making it difficult to achieve superior or even comparable low-frequency acoustic performance with a smaller back volume.

Porous materials such as glass fiber [8], melamine foam [9], etc., have been widely used as SAMs in the acoustic field. The pores, channels, or voids within SAMs enable them to absorb sound energy and effectively manage noise [10]. AEMs are a novel application in the acoustic field and need to be differentiated from SAMs. The SPL of a loudspeaker with porous material filling in the back volume is enhanced, instead of the sound energy being absorbed. J.R. Wright [11] put activated carbon into the back volume of a loudspeaker and successfully enhanced acoustic performance, especially in low frequencies. It was assumed that activated carbon would adsorb a portion of the compressed air in the back volume when it is subjected to vibration by the diaphragm. The lack of air molecules facilitates diaphragm vibration, and the compliance of the loudspeaker was improved, leading to better acoustic performance. R. Venegas et al. [12] investigated the sound absorption capacity of three activated carbons with different pore size distributions, finding that activated carbon with a higher proportion of micropores exhibited superior absorption capacity for low-frequency sounds attributed to the adsorption process of air molecules by nanometer size pores.

Zeolites are recognized as aluminosilicate crystals composed of TO_4_ tetrahedra (T generally represents Al or Si) with a consistent pore size. Adjustable pore size, acidity, and elemental composition make zeolite widely utilized in catalysis [13], separation [14] and ion exchange [15]. The uniform size of micropores in zeolite is a distinctive characteristic determined by the number of tetrahedra at the pore opening [16]. LTA zeolites have a pore size of approximately 0.4 nm with eight tetrahedra (8T) and MFI zeolite has approximately 0.5 nm pores with 10T [16]. The hydrophobicity of zeolite can be readily enhanced by increasing the Si/Al ratio [17] and electrical conductivity also decreases with lower aluminum contents [18]. Zeolite is a more promising commercial AEM for loudspeakers compared to activated carbon due to its uniform micropore size, as the micropore was considered to be the key factor in improving the low-frequency performance of activated carbon. Jiang et al. [19] improved the acoustic performance of a conventional microspeaker by 3.5 dB within the range of 100–1000 Hz by filling in the back volume with zeolite. Zhang et al. [2] prepared microspheres composed of SBA-15 and silica, used them to fill the back volume, and successfully reduced the resonance frequency of a microspeaker by more than 100 Hz. Jin Chen et al. [20] established a thermodynamic model of a compact loudspeaker filled with zeolite spheres, and discovered a linear relationship between mechanical compliance and the filling volume.

Maria Papakyriacou [21,22,23] has filed a series of patents and disclosed a loudspeaker design featuring zeolite-filled back volumes. This innovation significantly enhances bass performance, creating an effect equivalent to enlarging the back volume. To date, microspeakers in mobile phones continue to employ this method to improve acoustic performance. ZSM-5 zeolite with the MFI framework type has been integrated into billions of micro-loudspeakers, generating substantial business value.

ZSM-5 has been extensively researched as a catalyst due to its significant role in catalyzing petrochemical [24] and coal chemical processes [25]. ZSM-5 with MFI topology features a two-dimensional 10-member-ring crossed channel, a 10-member-ring straight channel along the (010) direction, and a 10-member-ring sinusoidal channel along the (100) direction, with dimensions of 0.53 nm × 0.56 nm and 0.51 nm × 0.55 nm, respectively [16]. Hierarchically structured ZSM-5 zeolites with micropores, mesopores, and macropores have been developed [26,27]. Christensen et al. [28] successfully synthesized MFI zeolite with a high mesoporous content using nano-sized carbon as a hard template, resulting in a significantly increased isobutane adsorption rate compared to traditional MFI zeolite. Johan C. Groen et al. [29] discovered that the adsorption rate of neopentane on desilicated low Si/Al ratio ZSM-5 zeolite with mesopores after alkaline treatment is 100 times higher than that of the untreated counterpart. The fast elution rate in ZSM-5 with mesopores, compared to purely microporous ZSM-5, was attributed to the enhanced transportation facilitated by the shortened diffusion path length of the auxiliary mesopore network [30].

Liu et al. [31] synthesized a composite material comprising zeolite and mesoporous silica, introducing adjustable mesopores to synergize with micropores in zeolites and effectively improve the low-frequency performance of micro-loudspeakers, proving diffusion and mass transport also play a crucial role in AEMs. Liu et al. [32] used ^129^Xe NMR spectroscopy to study the diffusion in hierarchically structured ZSM-5 and discovered that Xe exchange speed is much faster in mesoporous ZSM-5 than mechanically mixed microporous ZSM-5/mesoporous silica. Interior diffusion has been demonstrated to be more effective than physical mixing of microporous/mesoporous materials in adsorption/desorption processes. Therefore, hierarchically structured ZSM-5 holds great potential as an AEM.

The mechanism of AEMs remains under-researched, and the theory of SAMs is not applicable to AEMs. Air adsorption capacity was previously considered a key factor for zeolites to improve the *f*_0_ and SPL of microspeakers. However, our research revealed that 4A zeolite, which was believed to possess high nitrogen adsorption capacity [33], exhibited poor acoustic enhancement performance when tested in a nitrogen glovebox. This finding prompted us to question the conventional assumption that air adsorption capacity is the dominant factor. Instead, diffusion may play a more significant role than air adsorption in enhancing the *f*_0_ and SPL of microspeakers.

In this study, we synthesized hierarchically structured ZSM-5 zeolites with varying SiO_2_/Al_2_O_3_ ratios (SARs) and systematically investigated their morphology and pore structure. The acoustic enhancement performance of the zeolite specimens was evaluated based on the offset of resonance frequency (Δ*f*_0_) and SPL at 500 Hz (SPL@500Hz), as determined from the impedance curve and the resonance frequency curve. Our research has demonstrated that during the vibration of the speaker diaphragm, both micropores and supermicropores in zeolites can adsorb and desorb air molecules, whereas mesopores primarily enhance the diffusion of air molecules within the internal pore network of zeolites. Experimental results indicate that structures with a higher abundance of mesopores exhibit superior acoustic performance. Based on these findings, it is reasonable to speculate that interior diffusion plays a more critical role than adsorption capacity in determining the acoustic enhancement performance of zeolite.

## 2. Materials and Methods

### 2.1. Materials

Colloidal silica (30 wt%) was procured from Shandong Peak-tech New Material Co., Ltd. (Linyi, China). Aluminum sulfate octadecahydrate (Al_2_(SO_4_)_3_·18H_2_O), sodium hydroxide (NaOH), Tetrapropylammonium bromide (TPABr), were obtained from Aladdin Biochemical Technology Co., Ltd. (Shanghai, China), and were of analytical grade. Deionized water with a conductivity of <5 μS/cm was prepared by the lab water purification system.

#### Synthesis of Zeolite

In a typical synthesis, 3.33 g of Al_2_(SO_4_)_3_·18H_2_O was added into 520.13 g of deionized water and stirred until it was completely dissolved. Subsequently, 24.00 g of NaOH and 319.54 g of TPABr were gradually added into the above solution and stirred until they were dissolved. Finally, 801.12 g of 30% colloidal silica was incorporated at an extremely slow pace to prevent white flocculation. An additional 30 min stirring was conducted until a uniformly milk-white gel was achieved. The gel was transferred to a 2 L autoclave and carefully sealed. The autoclave was heated to 80 °C at a rate of 1 °C/min and maintained at 80 °C for 8 h, followed by heating to 150 °C with a heating rate of 1 °C/min, and held at 150 °C for 24 h. The entire crystallization process was conducted under stirring at a speed of 150 rpm. It was found that when the aging temperature was set at 80 °C, more crystal seeds were generated compared to aging at room temperature [34]. To achieve a better polycrystalline structure, the aging temperature was set at 80 °C.

The gel was taken out at a temperature below 50 °C after crystallization. The mother liquor was initially separated using a centrifuge at 3000 rpm for 5 min. The remaining filter cake was rinsed with deionized water multiple times until the conductivity of washed water was less than 100 μS/cm. Subsequently, the washed cake was dried at 120 °C for 12 h and then subjected to a calcination process in powder form with a specified temperature program using a muffle furnace. The muffle furnace underwent heating to 120 °C at a rate of 5 °C/min and maintained at that temperature for 120 min to remove residual water, followed by heating to 300 °C with a heating rate of 2 °C/min, and held at this temperature for 60 min; finally, the furnace was heated to 550 °C at a rate of 2 °C/min and maintained for 240 min. During the calcination process, it was observed that the template started to combust at approximately 300 °C. To avoid potential damage to the framework structure caused by the heat released during combustion, the calcination was held at 300 °C for 1 h to effectively remove the template agent at this stage. The heating rate was set to 2 °C/min to prevent the template from releasing excessive heat during combustion, which could lead to overheating and potentially destroy the zeolite structure.

The calcined powder was rinsed with deionized water multiple times until the conductivity of the washed water was less than 100 μS/cm. The resulting filter cake was then dried, pulverized, and subjected to calculation using the same temperature programs as mentioned above. The specimen was obtained after calculation and labelled as A_3_.

The molar ratio of A_3_’s prepared gel was as follows: SiO_2_:TPABr:H_2_O:Al_2_(SO_4_)_3_·18H_2_O:NaOH = 4:1.2:60:0.005:0.6. The preparation process for other samples with different SARs (SiO_2_/Al_2_O_3_ ratios) was identical and solely achieved by adjusting the amount of added Al_2_(SO_4_)_3_·18H_2_O. Five samples with different SARs were prepared, with SiO_2_/Al_2_O_3_ ratios of 100, 200, 800, 1600, and infinity, and were labeled as A_1_, A_2_, A_3_, A_4_, and A_5_, respectively.

### 2.2. Methods

#### 2.2.1. Characterization

X-ray diffraction (XRD) patterns were measured by a PANalytical X’Pert^3^ instrument with Cu Kα radiation (45 kV, 40 mA, λ = 0.15 nm) at a scanning rate of 8°/min from Malvern (Shanghai, China).

Nitrogen adsorption/desorption isotherms were measured at 77 K using the BELSORP-Max Nitrogen Physical Adsorption Instrument manufactured by Microtrac (Osaka, Japan). Prior to testing, samples were degassed at 573 K for 90 min. The surface area was calculated using the Braeuer–Emmett–Teller (BET) method, and the pore size distribution was calculated by the non-local density functional theory (NLDFT) method using desorption isotherms.

The morphology of zeolite samples was observed with a Regulus 8100 field-emission scanning electron microscope (FE-SEM) from HITACHI (Shanghai, China).

The SARs of the samples were measured using a ZSX Primus III+ X-ray fluorescence spectrometer (XRF) from Rigaku (Shanghai, China) operating at a tube voltage of 50 kV and tube current of 50 mA, employing semi-quantitative analysis. The measurement conditions were as follows: the measurement diameter was set to 30 mm, the Sample Auto-Selection Mode was disabled, the Optical Path Atmosphere was vacuum, the Component Type was oxide, the Target Material was Rh, the Crystal was LiF(200), and the Detector was SC.

Attenuated Total Refraction Fourier Transform Infrared spectroscopy (ATR-FTIR) spectra were recorded with a Nicolet iS5 instrument from Thermo Fisher Scientific (Shanghai, China).

The cross-sectional morphology of zeolite crystals was observed using an FEI Talos F200X G2 transmission electron microscope (TEM) from Thermo Fisher Scientific (Shanghai, China) with a 100 nm ultrathin section cut by an EM UC7 from Leica (Shanghai, China) using a diamond blade.

The nitrogen adsorption and desorption rate at 298 K were measured by a Dynamic Vapor/Gas Sorption Analyzer from BSD-DVS (Beijing, China). The samples were flushed by helium at 523 K for 180 min. The partial pressures (*P*/*P*_0_) tested were 95, 97.5 and 100.

The atomic force microscopy (AFM) images were taken in the tapping mode using an MFP-3D Infinity microscope from Asylum Research (Shanghai, China).

#### 2.2.2. Acoustic Performance Measurement

**Figure 1:**
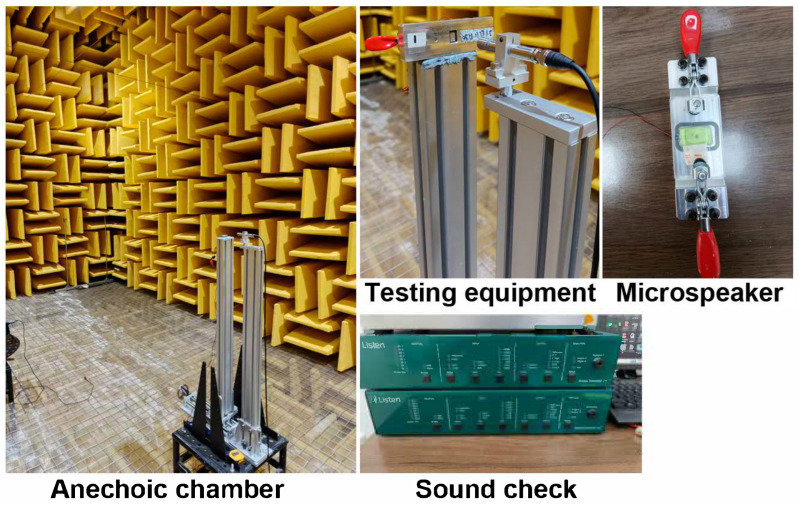
The test apparatus for measuring the impedance curve and frequency response curve.

The impedance curve and frequency response curve are critical characteristics of a loudspeaker, enabling the determination of the resonance frequency (*f*_0_) and sound pressure level (SPL@500Hz). The testing of these curves adheres to the international standard “IEC 60268-5 Sound system equipment—Part 5: Loudspeakers” [35]. The measurements were conducted using a Microspeaker 1115 with a 0.3 cm^3^ back volume, employing a SoundCheck system from Listen. The testing environment was maintained at a temperature of 23 ± 2 °C and within a relative humidity range of 40% to 70%, inside an anechoic chamber (Figure 1).

Initially, the impedance curve and frequency response curve were measured without any material in the back volume (Empty cavity), and *f*_01_ and SPL@500Hz_01_ were respectively obtained from the two curves. Subsequently, the resonance frequency *f*_02_ and SPL@500Hz_02_ were determined from the impedance curve and frequency response curve measured when a 0.06 g sample was added into the back volume. The acoustic performance of the sample was evaluated based on the offset in resonance frequency (Δ*f*_0_) and the increase in SPL@500Hz (ΔSPL@500Hz), which are calculated using the following formulas.

(1) $$∆f_{0}=f_{01}-f_{02}$$

(2) $$∆SPL@500Hz={SPL@500Hz}_{02}-{SPL@500Hz}_{01}$$

## 3. Results

### 3.1. Characterization Analysis

In order to investigate the impact of SAR on acoustic enhancement performance (Δ*f*_0_ and ΔSPL@500 Hz), the samples were synthesized with varying SARs of 87, 172, 614, 1233, and 6625, which were denoted as A_1_, A_2_, A_3_, A_4_ and A_5_, respectively. Table 1 summarizes the elemental compositions of the ZSM-5 zeolite samples, their corresponding SAR values, and their relative crystallinity. The actual SARs of ZSM-5 samples are notably influenced by the initial feed composition and are lower than the feeding SAR, which is in line with previous research findings [36,37]. This can be attributed to the consumption of aluminum, which serves as nucleation sites during ZSM-5 structure formation as reported by Kim [38,39], and the higher solubility of aluminum in alkaline solution compared to silicon facilitates its incorporation into the zeolite framework [40].

**Figure 2:**
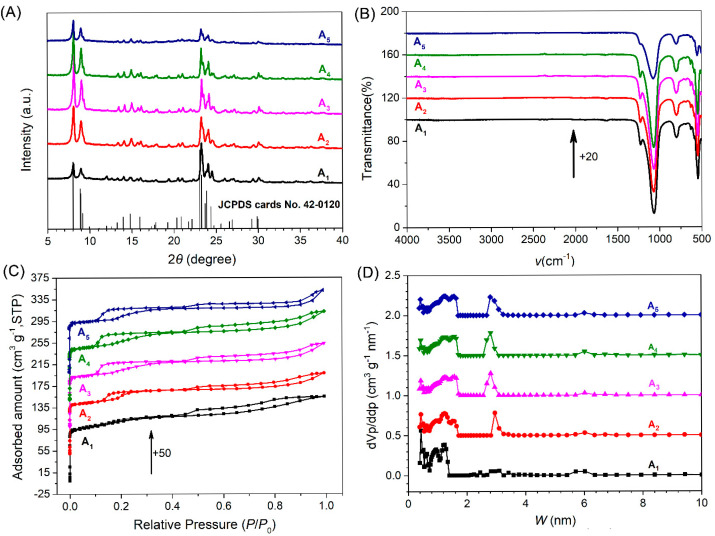
XRD pattern (**A**), IR spectra (**B**), nitrogen isotherms (**C**) and NLDFT pore size distribution (**D**) of ZSM-5 zeolites with different SARs of 87 (A_1_), 172 (A_2_), 614 (A_3_), 1233 (A_4_) and 6625 (A_5_).

Figure 2A shows the XRD patterns of samples with varying SARs. All samples exhibited a highly crystalline MFI topology structure, characterized by intense diffraction peaks at approximately 2*θ* = 7.9°, 8.7°, and three-pronged peaks between 23° and 24.5° [41]. No impurity peaks were detected when compared to the standard card (JCPDS card No. 42-0120), confirming that all samples were pure-phase ZSM-5 zeolites. The relative crystallinity was calculated based on the peak areas of the diffraction peaks at 2*θ* = 7.9°, 8.7°, and between 23° and 24.5°. Sample A_1_, with the lowest SAR of 87, displayed the highest intensity for the diffraction peaks in the range of 23° to 24.5°, while the two peaks at 7.9° and 8.7° showed the lowest intensity. As the SAR increased from 87 (A_1_) to 1233 (A_4_), the intensity of the diffraction peaks at 7.9° and 8.7° progressively strengthened, whereas the intensity of the peaks in the range of 23° to 24.5° slightly weakened. It is observed that A_5_, with the highest SAR of 6625, exhibited the lowest relative crystallinity based on the intensities of the five characteristic peaks in the XRD patterns.

In contrast to samples with low SAR (A_1_ and A_2_), the A_3_, A_4_, and A_5_ samples exhibited doublet diffraction peaks at approximately 2*θ* = 24.4° and 29.2° in their XRD patterns, indicative of monoclinic symmetry in their crystal system (as shown in Figure 2A). Notably, ZSM-5, synthesized with a high SAR, tended to transition from orthorhombic to monoclinic symmetry following the removal of organic matter through calcination [42].

The relative crystallinity of A_1_ and A_2_ was lower than that of A_3_ and A_4_, particularly in the diffraction peaks at 2*θ* = 7.9 and 8.7°. The diffraction peak at 7.9° represents the (101) crystal plane, while the peak at 8.7° corresponds to the (020) crystal plane and straight channel of the MFI structure [43].

The IR spectra in Figure 2B also reveal characteristic bands of the MFI topological structure, with bands around 550, 800, 1100 and 1250 cm^−1^ [44]. The band at approximately 1250 cm^−1^ corresponds to the asymmetrical stretching vibration of the external T-O bond. The band at around 1100 cm^−1^ is associated with the asymmetrical stretching vibration of the internal T-O bond. The band at approximately 800 cm^−1^ is attributed to the symmetrical stretching vibration of the T-O bond [45]. In the IR spectra of A_1_, A_2_, A_3_, and A_4_, the intensities of the four absorption bands are nearly identical, and the intensities of the three-pronged peaks in the 23°~24.5° range in their XRD patterns also shows similarity. However, A_5_, with an SAR of 6625, exhibits the lowest intensity of the three characteristic peaks in its XRD pattern and displays the weakest intensities at approximately 550, 800, 1100, and 1250 cm^−1^ in its IR spectra, indicating that the relative crystallinity of the samples was strongly correlated with the intensity of the characteristic bands in their IR spectra.

The nitrogen adsorption–desorption isotherms in Figure 2C demonstrate that all samples exhibited curves combined with type-I and type-IV isotherms, suggesting the co-existence of micropores and mesopores [46]. Sample A_1_ shows a single hysteresis loop, while the other samples display two hysteresis loops at partial pressures (*P*/*P*_0_) in the range of 0.1~0.4, and 0.4~1.0, respectively. Hysteresis loops are commonly observed in the range of *P*/*P*_0_ = 0.4~1.0 in hierarchical ZSM-5, indicating the presence of abundant mesopores within the crystals and capillary condensation occurring in these mesopores, as reported by previous studies [36,47,48].

Hysteresis loops between *P*/*P*_0_ = 0.1~0.4 are commonly referred to as “low pressure hysteresis loops” and have been associated with a fluid-to-crystalline phase transition of the adsorbed nitrogen [49]. The presence of low pressure hysteresis loops has been linked to pores with sizes around 2 nm, which are termed “supermicropores” [50]. Analysis of the pore size distribution in Figure 2D reveals that all samples possessed supermicropores with sizes ranging from 1 to 2 nm. It can be deduced that the size of the low-pressure loop is strongly correlated with the supermicropores centered at approximately 1.7 nm. No supermicropores near this size were observed in A_1_, and the quantity of supermicropores around 1.7 nm in A_2_ was less than that in A_3_, A_4_, and A_5_. Additionally, apart from the supermicropores, micropores with a size of approximately 0.5 nm characteristic of conventional ZSM-5, as well as mesopores with sizes of approximately 2.8 nm and 6.0 nm, were also observed in the pore size distribution of all samples.

**Figure 3:**
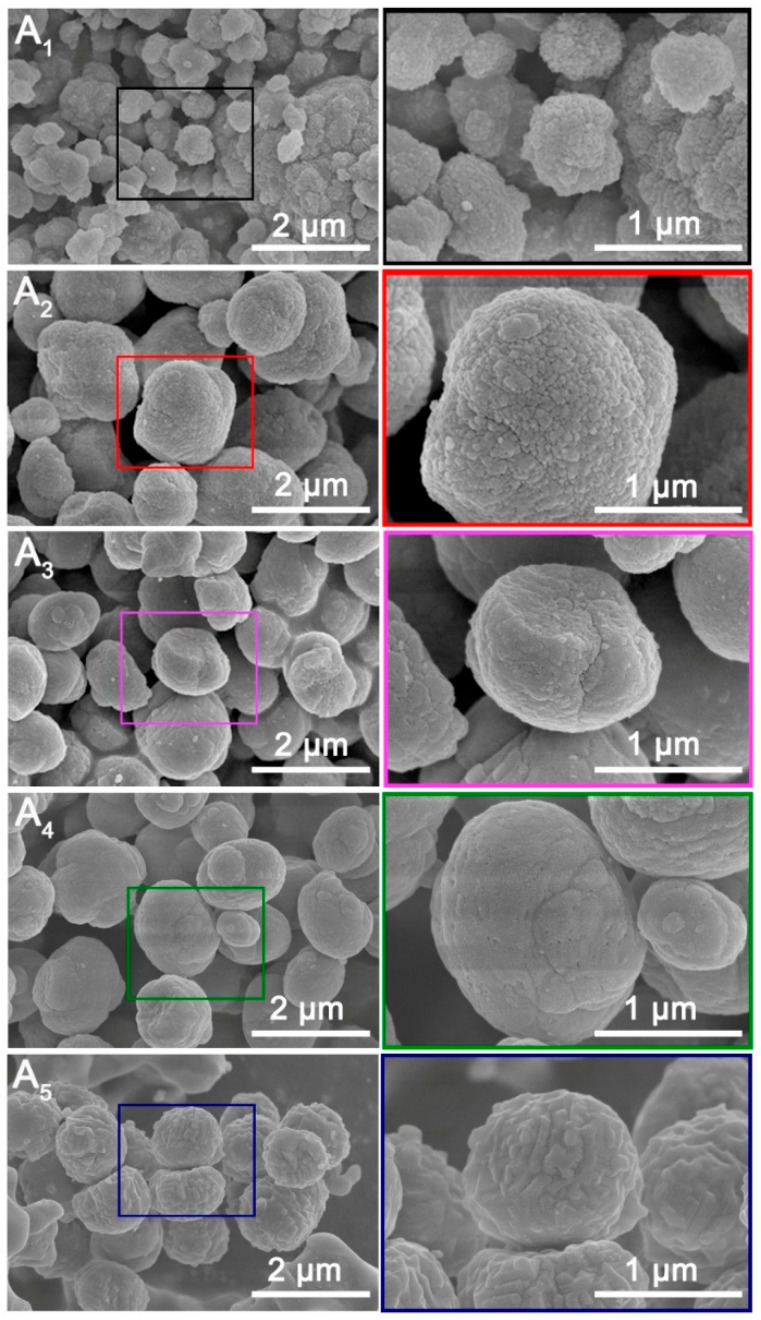
SEM images of ZSM-5 samples with different SAR.

The SEM images in Figure 3 reveal that all samples consisted of secondary agglomerates formed from MFI nanocrystals with rough surfaces. It is unsurprising that micro-sized, sphere-shaped ZSM-5 aggregates were obtained when TPABr was used as the structure-directing agent [51]. The size distribution of the grains can be found in Figure S1. Differentiating the shape of A_1_ crystals is challenging due to their broad size range, primarily spanning 440~588 nm. As the SAR increases to 172 (A_2_), 614 (A_3_), 1233 (A_4_), and 6624 (A_5_), the granules become more regular and spherical in shape. Specifically, the size of A_2_’s microspheres ranged from 1371~1846 nm, A_3_’s from 1057~1231 nm, A_4_’s from 1004~1279 nm, and A_5_’s from 1075~1253 nm.

The surface roughness measured by AFM can be found in Table S1. As the SAR increases, the surface of the granule becomes smoother. The difference in roughness can be attributed to the aluminum content of the gel. Aluminum requires sodium hydroxide for dissolution. Thus, if the gel contains the same amount of sodium hydroxide, the higher the aluminum content, the lower the alkalinity. Low alkalinity leads to high supersaturation, and at high supersaturation, nucleation occurs simultaneously at multiple places, resulting in a rough crystal surface [52].

**Figure 4:**
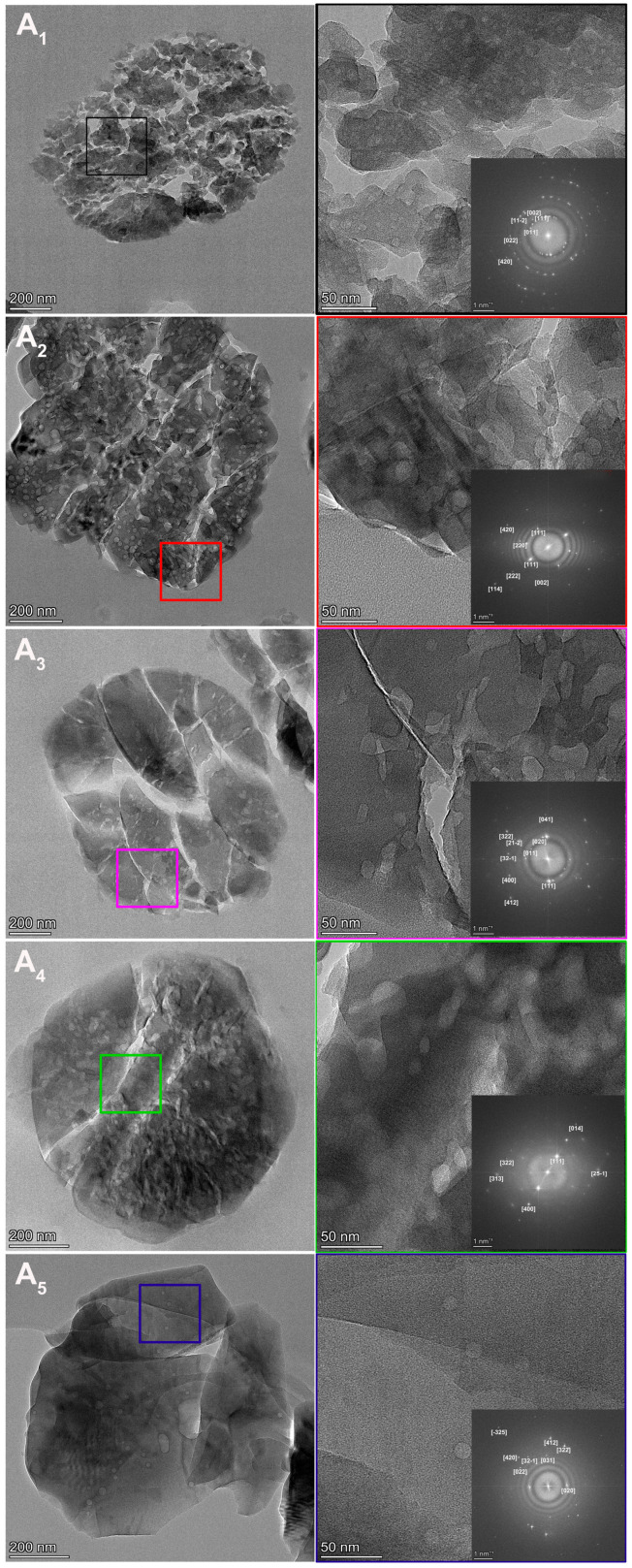
Cross-sectional TEM images and their corresponding FFT patterns of ZSM-5 samples with different SARs.

Interior cross-sectional images of ZSM-5 agglomerates with varying SARs are depicted in Figure 4. Smaller particles were selected to enhance the precision of observation. In the low-resolution images, the square regions were magnified to provide a clearer observation of the pore structure via high-resolution imaging. The insets show the corresponding Fast Fourier transform (FFT) patterns. The TEM findings are in accordance with the SEM image, indicating that all samples were agglomerates of multiple MFI nanocrystals. The size of A_1_’s nanocrystals ranged from 42 to 115 nm, and that of A_2_ from 100 to 200 nm. The width of A_3_’s nanocrystals was approximately 200 nm, and the length reached 500 to 700 nm. It was extremely difficult to determine the size of A_4_’s nanocrystals, as the boundaries were blurred; the size is approximately 400 to 600 nm. The aggregate of A_5_ consisted of only a few large crystals, and the largest size was approximately 600 to 700 nm.

The crystallization process of ZSM-5 can be categorized into two distinct stages: nucleation and crystallization [53]. In gel A_1_, the highest supersaturation level among the experimental samples facilitated the formation of the highest density of nuclei, whereas a higher Si/Al ratio reduces supersaturation, resulting in fewer nuclei. Notably, aluminum ions are widely recognized to inhibit the crystallization process [48]. Consequently, as sample A_1_ had the slowest crystallization kinetics, the nuclei developed into relatively small nanocrystals. The size of nanocrystals exhibits a positive correlation with SAR. Furthermore, nanocrystals exhibit a thermodynamic tendency to minimize surface energy through agglomeration, as confirmed by SEM and TEM observations [49].

The well-defined lattice fringes observed in A_1_’s nanocrystals demonstrated pronounced crystallinity, corroborating the structural characteristics identified by XRD and FTIR analyses. The varying crystallographic orientations of these fringes confirmed the polycrystalline nature of A_1_, further evidencing its aggregated morphology. This conclusion is reinforced by the inset FFT image, which reveals a polycrystalline structure characterized by concentric diffraction rings generated by discrete crystallites. Notably, mesopores with diameters in the nanoscale range were consistently distributed throughout the nanocrystals, as resolved by high-resolution TEM imaging.

A_2_ exhibited a tighter structural organization compared to A_1_. The lattice fringes are clearly visible in the high-resolution image, showing a higher degree of orientation consistency than in A_1_. The primary distinction between A_2_ and A_1_ lies in the mesopores, which are characterized by their irregular shapes and sizes, as observed in TEM images. The TEM image of A_2_ reveals two types of mesopores: slits and gaps between nanocrystals, which can also be observed by SEM, and voids within crystals resembling round or cylindrical shapes. These mesopores are notably larger than those in A_1_.

The structure of A_3_ was similar to that of A_2_, and both types of mesopores are also present in the TEM image of A_3_. Compared to A_2_, the mesopores within the crystals exhibited a similar size range. The TEM image of A_4_’s spheres shows a more pronounced spherical and intact morphology compared to A_2_ and A_3_, as evidenced by the blurred boundaries between nanocrystals. Developed mesopores within the crystals can also be observed. The A_5_ TEM image reveals the most compact structure, consistent with observations from the SEM image. No slits or gaps are present. Mesopores within the crystals are also visible, albeit in smaller quantities compared to other samples.

As shown in Table 2, all samples exhibited similar surface areas, ranging from 382 to 394 m^2^/g, and similar pore volumes, within the range of 0.23~0.25 cm^3^/g. However, there is a notable discrepancy in external surface area and micropore volume. A_1_, which had the lowest SAR, demonstrated the largest micropore volume of 0.19 cm^3^/g and the smallest external surface area of 31 m^2^/g. Its greater micropore volume can be attributed to its smaller nanocrystals, as it possessed the greatest number of micropores around 0.5 nm in the pore size distribution. The mesopore volume of A_1_ was the lowest because the mesopore size within its nanocrystals was smaller compared to that of other samples. This is also evident in the pore size distribution, as A_1_ had the fewest mesopores around 2.8 nm.

The nanocrystal size of A_2_ was larger than that of A_1_, and the mesopores within its crystals were also larger, resulting in a decrease in micropore volume from 0.19 to 0.14 cm^3^/g and an increase in external surface area from 31 to 47 m^2^/g. A_3_ exhibited a larger external surface area of 60 m^2^/g compared to A_2_, along with a smaller micropore volume of 0.12 cm^3^/g. A_4_ had the largest external surface area of 68 m^2^/g. When the SAR increased to 6624 (A_5_), the external surface area decreased back to 50 m^2^/g, which can be attributed to that sample’s tighter structure.

### 3.2. Acoustic Enhancement Performance

**Figure 5:**
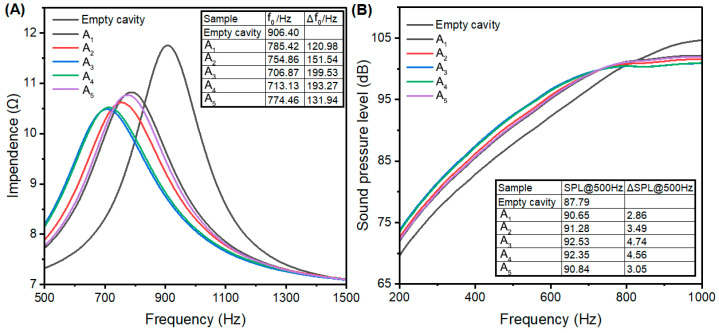
Impendence curve (**A**) and frequency response curve (**B**) of ZSM-5 samples with different SARs.

The impedance curve and frequency response curve of ZSM-5 samples with varying SARs are presented in Figure 5. As shown in Figure 5, the resonance frequency offset (Δ*f*_0_) and the increase in sound pressure level at 500 Hz (ΔSPL@500Hz) are partially correlated with the SAR values. Sample A_1_, with the lowest SAR of 87, exhibited the weakest performance, with a Δ*f*_0_ of 120.98 Hz and a ΔSPL@500Hz of 2.86 dB. As the SAR increased from 87 (A_1_) to 172 (A_2_), the Δ*f*_0_ increased to 151.54 Hz and the ΔSPL@500Hz rose to 3.49 dB. Samples A_3_ and A_4_, with SAR values of 614 and 1233, demonstrated superior performance, achieving Δ*f*_0_ values of 199.53 Hz and 193.27 Hz, and ΔSPL@500Hz values of 4.74 dB and 4.56 dB, respectively. Conversely, sample A_5_, with the highest SAR, showed relatively poor acoustic enhancement performance, with a Δ*f*_0_ of 131.94 Hz and a ΔSPL@500Hz of 3.05 dB.

AEMs are always related to sound-absorbing materials (SAMs), and many people conflate the two types of materials because they are both porous and employed in the acoustic field. Nevertheless, their applications are entirely different. SAMs are employed to reduce noise and vibration, and the main objective of SAMs is to lower the reverberant sound pressure level [10]. In contrast, AEMs are employed to enhance the sound pressure level of microspeakers. When sound waves enter SAMs, the air molecules on the surface and within the pores of the material are compelled to vibrate. The sound energy is mainly dissipated through frictional heating between the vibrating air molecules and the pore surface. Sound energy is absorbed by SAMs during the multiple reflections of the sound wave, and small pores are considered to result in less sound attenuation. Therefore, foams with appropriate pore sizes are typically utilized as SAMs [54]. The mechanism of SAMs cannot explain why the acoustic performance of a microspeaker is improved by filling in the back volume if the sound energy is adsorbed by porous materials.

The acoustic enhancement performance of traditional SAMs, such as commercial sound adsorbing cotton made of melamine used in mobile phones, has been tested with the same method (Figure S3), and the resulting Δ*f*_0_ values were merely 104.29 and 106.02 Hz, and ΔSPL@500Hz of 2.45 and 2.52 dB. These results lead us to believe that the microporous structure of zeolite has an advantage when utilized as an AEM compared to melamine foams, and the mechanism of AEMs is clearly different from that of SAMs.

The resonance frequency $f_{0}$ of microspeakers can be calculated using Formula (3) [55], where M represents the mechanical dynamic mass of the loudspeaker, $K_{total}$ denotes the total stiffness of the system and can be calculated using Formula (4) [55], while $K_{unit}$ represents the unit stiffness, S denotes diaphragm area, $\rho_{0}$ denotes air density in back volume and c denotes sound velocity in air, while $V_{cc}$ denotes the back volume. Increasing the back volume is a key factor in decreasing resonance frequency according to this formula. In the test system discussed in this paper, $K_{unit}$ and S are constants, with 0.06 g of zeolite filling in the back volume, so M can also be considered as a constant while the mechanical dynamic mass of the test system is 52 g. The actual back volume is less than 0.3 cc due to space reduction occupied by zeolite filling. If the zeolite filling does not play any part in the back volume, the resonance frequency will increase due to the reduction of the back volume. The porous filling material in the back volume certainly plays a crucial role, as evidenced by the reduction in resonance frequency ($f_{0}$).


(3) f0=12πKtotalM

(4) Ktotal=Kunit+S2ρ0c2Vcc

Sound waves are generated by the vibration of the diaphragm, causing a change in pressure within the enclosed back volume. When the diaphragm vibrates in the direction of the back volume, the pressure increases due to the shrinking volume according to the Ideal Gas Equation [56], and air molecules are adsorbed into the pores of the porous material which fills in the enclosure due to increased pressure. The reduction of air molecules in the back volume makes it easier for the diaphragm to vibrate under the same force. A larger vibration amplitude results in a louder sound. So, the adsorption capacity of air was thought to be crucial to AEMs [19].

SAMs absorb sound energy while AEMs adsorb air molecules. This results in different requirements for pore size. The sound pressure is very low, with a reference sound pressure of only 20 mPa [57]. Micropores of zeolite exhibit sensitivity to adsorbing nitrogen at very low partial pressures, as indicated by the nitrogen adsorption isotherms shown in Figure 2C. With a molecular kinetic diameter of nitrogen at 0.364 nm and oxygen at 0.346 nm [58], the smaller-size pores in zeolite tend to adsorb smaller molecules due to reinforced van der Waals interactions [59]. The pore size of straight and sinusoidal channels in ZSM-5 is approximately 0.55 nm [16] and is sensitive to adsorption of nitrogen and oxygen molecules under sound pressure. Conversely, the pore size of melamine foam ranges from 50 to 500 μm, which might be suitable as a SAM to absorb sound energy, but makes it difficult to adsorb air molecules, resulting in poor performance.

The human-perceivable sound frequency ranges from 20 to 20,000 Hz [60], corresponding to a time frame of 0.05 to 0.00005 s, indicating that the air adsorption-desorption process should be completed within 0.05 s. However, due to the rapid nature of this process, there is insufficient time for air molecules to reach the inner space of the channel, particularly at intersections between straight and zigzag channels. As a result, cyclic adsorption-desorption only occurs in the vicinity of micropore openings, thus rendering most of A_1_’s micropore volume ineffective during split-second adsorption-desorption processes. Therefore, it is not micropore volume or surface area but rather the number of micropores accessible by air molecules that determines air adsorption-desorption during instantaneous oscillation of the diaphragm in microspeakers.

To achieve superior acoustic enhancement performance, zeolite must have a specific number of micropores slightly larger than air molecules that can be accessed by air in a split-second. It is evident that smaller crystals result in more micropores compared to larger ones due to their large surface area. However, nanocrystals tend to agglomerate and form micro-sized aggregates due to high surface Gibbs energy. The introduction of mesopores such as intracrystalline voids in hierarchically structured ZSM-5 is necessary for rapid access of air molecules to the micropores. The incorporation of mesopores in the hierarchical structure significantly enhances diffusion and mass transport in ZSM-5 [28,61].

The XRD and FTIR results confirm similar crystallinity for all samples except A_5_, while the surface area and pore volume remained nearly identical across all samples. However, significant variations in mesopore volume, micropore volume, and external surface area substantially influenced acoustic enhancement performance. Samples A_3_ and A_4_ exhibited the highest mesopore volume (0.12 cm^3^/g) and demonstrated superior performance with resonance frequency offsets of 199.53 Hz and 193.27 Hz, respectively, and sound pressure level increases of 4.74 dB and 4.56 dB, respectively. In contrast, sample A_1_, despite having the highest micropore volume and surface area but the lowest mesopore volume, exhibited the poorest performance with a resonance frequency offset of only 120.98 Hz and a sound pressure level increase of merely 2.86 dB.

This clearly indicates a strong correlation between acoustic enhancement performance and mesopore volume, suggesting that micropores alone may not be decisive factors in acoustic enhancement. The poor performance of A_1_ can be attributed to its limited external surface area and smaller mesopore size, which result in slower air diffusion rates compared to other samples. A remarkable improvement in acoustic enhancement performance was observed with the progressive increase in external surface area from 47 m^2^/g (A_2_) to 60 m^2^/g (A_3_) and 68 m^2^/g (A_4_).

Notably, there was little difference between A_2_ and A_3_ in terms of mesopores centered at approximately 2.8 nm and 6.0 nm. However, A_3_ contained a significantly higher quantity of supermicropores centered at ~1.7 nm compared to A_2_, indicating that supermicropores play a critical role in enhancing acoustic performance.

The presence of gaps and slits between nanocrystals is also crucial for acoustic enhancement as they enable air molecules to penetrate into the crystal interior more effectively. Sample A_5_, despite exhibiting the highest SAR, demonstrated poor acoustic enhancement performance with a resonance frequency offset of only 131.94 Hz and a sound pressure level increase of merely 3.05 dB. This can be attributed to its tightly packed structure, as evidenced by TEM analysis, which limited the formation of mesopores and reduced air accessibility.

The nitrogen adsorption and desorption rates of A_2_, A_3_, and A_4_ at 298 K were determined using a dynamic sorption analyzer. The adsorption capacity versus time curve is presented in Figure S4A. At 298 K, the order of nitrogen adsorption capacity was A_2_ > A_3_ > A_4_. The adsorption rate was calculated based on the slope between two points: the adsorption capacity at the final point when *P*/*P*_0_ = 0.975 and the adsorption capacity at the initial point when the partial pressure increased from 0.975 to 1.0. The slopes for A_2_, A_3_, and A_4_ are 0.0444, 0.0557, and 0.0557 (Figure S4B), respectively. Thus, the order of adsorption rate was A_3_ = A_4_ > A_2_. The desorption of nitrogen when the partial pressure decreased from 1.0 to 0.975 was relatively insensitive, as shown in Figure S4C. The desorption rate was determined by the slope between four points: the adsorption capacity at the final point when *P*/*P*_0_ = 1.0 and the adsorption capacities at the first three points when *P*/*P_0_* = 0.975. The desorption slopes for A_2_, A_3_, and A_4_ were −0.0268, −0.0225, and −0.0269, respectively. It can be inferred that the adsorption rate of nitrogen has a strong correlation with the acoustic enhancement performance of AEMs.

### 3.3. Impact of Sodium Content

Apart from pore structure, the quantity of residue cations may also contribute to the poor performance of low SAR samples. The presence of residual sodium pieces in the channels, whether in micropores or mesopores, hinders the propagation of air molecules and negatively affects acoustic enhancement performance. To investigate the effect of the quantity of cations on the acoustic enhancement of ZSM-5 zeolite, during the preparation of A_3_, three specimens of filter cake were taken out at different stages of the rinsing process after the first crystallization. Specifically, the conductivity of the washed water was measured as 850 μS, 650 μS, and 298 μS, respectively. The three filter cakes were subsequently subjected to the same drying, pulverizing, and calcination steps as mentioned above. The resulting samples were labeled as A_3_-Na-1, A_3_-Na-2, and A_3_-Na-3.

The sodium content and textural properties of the three samples can be found in Table 3. As anticipated, the sodium content exhibited a decreasing trend in parallel with the reduced conductivity of the washing water, and the sodium contents of A_3_-Na-1, A_3_-Na-2, and A_3_-Na-3 were 1.04%, 0.68%, and 0.53%, respectively. A significant variation was observed in the surface area and pore volume among the three specimens as a function of sodium content. Specifically, A_3_-Na-1 exhibited a surface area of 430 m^2^/g and a total pore volume of 0.25 cm^3^/g, with micropore volume contributing 0.21 cm^3^/g. As the sodium content decreased, both the surface area and micropore volume decreased progressively. For A_3_-Na-2 and A_3_-Na-3, the surface areas were 400 m^2^/g and 378 m^2^/g, respectively, while the micropore volumes were 0.17 cm^3^/g and 0.13 cm^3^/g, respectively.

**Figure 6:**
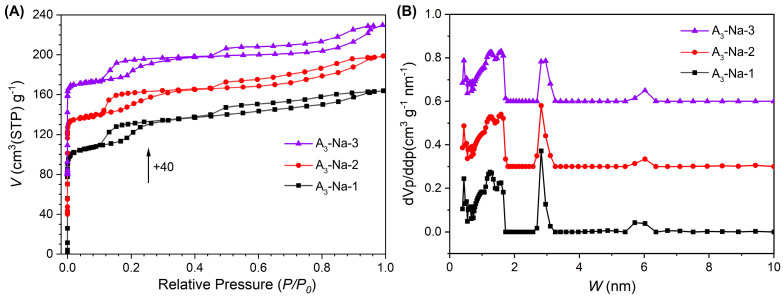
Nitrogen isotherms (**A**) and NLDFT pore size distribution (**B**) of A_3_ samples with different sodium contents.

Nitrogen isotherms and pore size distributions for the three samples are depicted in Figure 6. All isotherms resemble that of A_3_, with both hysteresis loops clearly evident. Minor differences exist in the pore size distribution: the quantity of supermicropores centered at ~1.7 nm in A_3_-Na-1 is lower than in A_3_-Na-2 and A_3_-Na-3, while the mesopore size around ~2.8 nm increases progressively as sodium content decreases. This suggests that residual sodium ions predominantly occupy supermicropores and mesopores.

**Figure 7:**
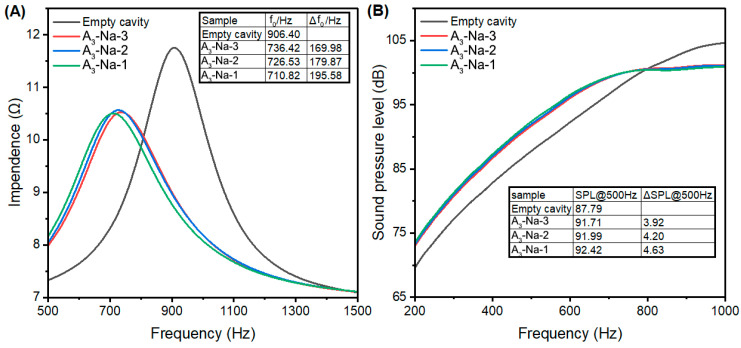
Impendence curve (**A**) and frequency response curve (**B**) of A_3_ zeolites with different sodium contents.

The impedance curves and frequency response curves of ZSM-5 samples with varying sodium contents are shown in Figure 7. The Δ*f*_0_ values of A_3_-Na-1, A_3_-Na-2, and A_3_-Na-3 were 169.98 Hz, 179.87 Hz, and 195.58 Hz, respectively, while the ΔSPL@500Hz values were 3.92 dB, 4.20 dB, and 4.63 dB, respectively. Despite A_3_-Na-1 possessing the highest surface area and micropore volume, its performance was inferior due to higher sodium residue. Conversely, A_3_-Na-3, which exhibited the lowest total surface area but the largest external surface area, demonstrated superior acoustic performance, likely attributed to reduced internal blockage by sodium cations.

### 3.4. Impact of Pore Structure

**Figure 8:**
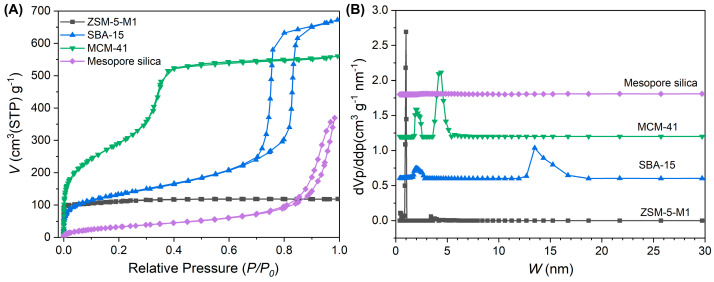
Nitrogen isotherms (**A**) and NLDFT pore size distribution (**B**) of porous materials with different pore structures.

**Figure 9:**
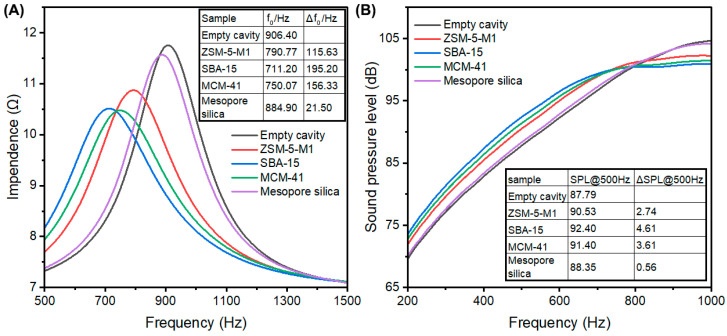
Impendence curve (**A**) and frequency response curve (**B**) of porous materials with different pore structures.

A variety of porous materials with distinct pore structures were selected to systematically investigate the influence of pore structure on acoustic enhancement performance. Detailed sample information and their textural properties are summarized in Table 4. Nitrogen adsorption–desorption isotherms along with corresponding pore size distributions are illustrated in Figure 8, while impedance spectra and frequency response curves are presented in Figure 9.

ZSM-5-M1 exhibited a Type I isotherm with negligible mesopore contributions in the pore size distribution. Despite its high surface area of 418 m^2^/g and the presence of micropores and supermicropores, it demonstrated suboptimal performance with Δ*f*_0_ = 115.63 Hz and ΔSPL@500Hz = 2.74 dB. This suggests that the absence of mesopores significantly compromises acoustic performance, indicating that zeolites lacking mesopores are less favorable for this application.

SBA-15 had a surface area of 466 m^2^/g and displays a pronounced hysteresis loop in its nitrogen isotherm between *P*/*P*_0_ = 0.7 ~ 1.0, corresponding to mesopore sizes ranging from 10 to 20 nm. Additionally, supermicropores centered at approximately 1.96 nm were observed. MCM-41, with a surface area of 245 m^2^/g, featured mesopores around 4.40 nm and supermicropores around 1.96 nm. Mesoporous silica, in contrast, exhibited a relatively low surface area of 125 m^2^/g and lacked micropores entirely.

The Δ*f*_0_ values of SBA-15, MCM-41, and Mesoporous silica are 195.2 Hz, 156.33 Hz, and 21.50 Hz, respectively, while their ΔSPL@500Hz values are 4.61 dB, 3.61 dB, and 0.56 dB, respectively. The inferior performance of Mesoporous silica underscores the necessity of micropores for efficient adsorption and desorption of air molecules under minor pressure changes. The superior performance of SBA-15 and MCM-41 highlights the critical role of supermicropores in enhancing acoustic performance, likely enabling more efficient molecular adsorption during microspeaker operation.

## 4. Conclusions

This study systematically investigates the influence of pore structure on the acoustic enhancement performance of ZSM-5 zeolite when utilized as an AEM. Hierarchically structured ZSM-5 samples with varying nanocrystal aggregate morphologies were synthesized via the hydrothermal method under different SAR conditions. As the SAR increased, the supersaturation of the gel decreased due to reduced aluminum content, resulting in distinct changes in crystal morphology and pore structure. ZSM-5 samples with high SAR exhibited abundant mesopores and supermicropores.

The acoustic enhancement performance of zeolites depends more on their pore structure than on air adsorption capacity, with mesopores and supermicropores playing critical roles. Specifically, ZSM-5 samples with SAR values of 614 and 1233, featuring micropores, supermicropores, and mesopores, demonstrated exceptional acoustic enhancement performance, achieving Δ*f*_0_ values of 199.53 Hz and 193.27 Hz, and ΔSPL@500Hz values of 4.74 dB and 4.56 dB, respectively. The presence of supermicropores significantly enhances acoustic performance by enabling AEMs to efficiently adsorb and desorb air molecules under minor pressure changes.

Our research challenges the conventional perception of AEMs, distinguishing them from SAMs, and provides a valuable guideline for researchers developing advanced AEMs. This work highlights the importance of hierarchical pore structures in optimizing acoustic enhancement performance.

## Figures and Tables

**Table 1:** Elemental compositions of the ZSM-5 zeolite samples, their corresponding SAR values, and their relative crystallinity.

Sample	Na_2_O (wt%)	Al_2_O_3_ (wt%)	SiO_2_ (wt%)	Actual SAR	Relative Crystallinity (%)
A_1_	1.53	1.88	96.29	87	76
A_2_	0.64	0.97	98.10	172	97
A_3_	0.22	0.28	99.31	614	100
A_4_	0.20	0.14	99.35	1233	73
A_5_	0.17	0.03	99.59	6625	30

**Table 2:** Textural properties of ZSM-5 samples with different SARs.

Sample	*S*_total_ (m^2^·g^−1^)	*V*_total_ (cm^3^·g^−1^)	*S*_ext_ (m^2^·g^−1^)	*V*_micro_ (cm^3^·g^−1^)	V_meso_ (cm^3^·g^−1^)
A_1_	394	0.24	31	0.19	0.05
A_2_	384	0.23	47	0.14	0.09
A_3_	384	0.24	60	0.12	0.12
A_4_	389	0.25	68	0.13	0.12
A_5_	382	0.23	50	0.13	0.10

**Table 3:** Sodium content and textural properties of A_3_ zeolites with different sodium contents.

Sample	Na (wt%)	*S*_total_ (m^2^·g^−1^)	*V*_total_ (cm^3^·g^−1^)	*S*_ext_ (m^2^·g^−1^)	*V*_micro_ (cm^3^·g^−1^)	*V_meso_* (cm^3^·g^−1^)
A_3_-Na-1	1.04	430	0.25	27	0.21	0.04
A_3_-Na-2	0.68	400	0.25	47	0.17	0.08
A_3_-Na-3	0.53	378	0.23	57	0.13	0.10

**Table 4:** Sample information, textural properties and acoustic enhancement performance of porous materials with different pore structures.

Sample	*S*_total_ (m^2^·g^−1^)	*V*_total_ (cm^3^·g^−1^)	*S*_ext_ (m^2^·g^−1^)	*V*_micro_ (cm^3^·g^−1^)	*V_meso_* (cm^3^·g^−1^)
ZSM-5-M1	418	0.18	0	0.18	0
SBA-15	466	1.04	70	0.91	0.13
MCM-41	245	0.87	19	0.83	0.04
Mesoporous silica	125	0.57	125	0	0.57

## Data Availability

Data are contained within the article.

## Supplementary Materials

The following supporting information can be downloaded at: https://www.mdpi.com/article/10.3390/nano15110797/s1, Figure S1: Size distribution of ZSM-5 grains in Figure 2 ((**A**) A_1_, (**B**) A_2_, (**C**) A_3_, (**D**) A_4_, (**E**) A_5_); Figure S2: AFM images of ZSM-5 with different SAR. A_1_ (**A**), A_2_ (**B**), A_3_ (**C**), A_4_ (**D**), A_5_ (**E**); Figure S3: Impendence curve and frequency response curve of sound-absorbing cotton: (**A**) MF-1; (**B**) MF-2; Figure S4: Adsorption capacity-Time curve (**A**), adsorption rate when *P*/*P_0_* increase from 0.975 to 1.0 (**B**), desorption rate when *P*/*P*_0_ decrease from 1.0 to 0.975 (**C**). Table S1: Roughness of ZSM-5 with different SARs. Table S2: Detailed elemental compositions of ZSM-5 zeolite samples with different SARs.
