# Supplementary material for: Acoustic Enhancement Performance of Hierarchical ZSM-5 Zeolites with Different Si/Al Ratios
Source: Nanomaterials (Basel). 2025 May 26;15(11):797. doi: 10.3390/nano15110797 (PMC12157734; doi:10.3390/nano15110797)
Supplement: Supplementary file 1 [file nanomaterials-15-00797-s001.zip › nanomaterials-3645849-supplementary 1.pdf]

Supplementary data

Acoustic Enhancement Performance of hierarchical ZSM-5 Zeolites with different Si/Al ratios

Guo Mingbo[1,2], *Wang Yijun*[2], *Zhang Lei*[2], *Lu Junran*[2], *Gong Chang*[2], *Wanning Zhang*[1], *Yuxi Fang*[1], *Zhu Xinyuan*[1*], *Che Shunai*[1*]

1. State Key Laboratory of Synergistic Chem-Bio Synthesis, School of Chemical Science and Engineering, Frontiers Science Center for Transformative Molecules, Shanghai Key Laboratory for Molecular Engineering of Chiral Drugs, Shanghai Jiao Tong University, 800 Dongchuan Road, Shanghai, 200240 P. R. China.

2. SSI New Material (Zhenjiang) Co.,Ltd., 7 Songlin Mountain Road, Zhenjiang, 212006 P. R. China.

Materials

ZSM-5-M1：ZSM-5 zeolite with a SAR of 600 as specified, purchased from Zhuoyue Environmental Protection New Materials (Shanghai) Co., Ltd. (Shanghai, China)

SBA-15: Pure silica SBA-15 zeolite purchased from Zhuoyue Environmental Protection New Materials (Shanghai) Co., Ltd. (Shanghai, China)

MCM-41: Pure silica MCM-41 zeolite purchased from Dalian ZR Catalyst material Co., Ltd. (Liaoning, China)

Mesopore silica: Silica of mesopore as specified purchased from Hangzhou Jiayou New Material Co., Ltd. (Zhejiang, China)

MF-1: Sound adsorbing cotton, purchased from Shanghai Junhua New Material Co., Ltd. (Shanghai, China)

MF-2: Sound adsorbing cotton, purchased from Guangdong Tiange Acoustic Material Co., Ltd. (Guangdong, China)

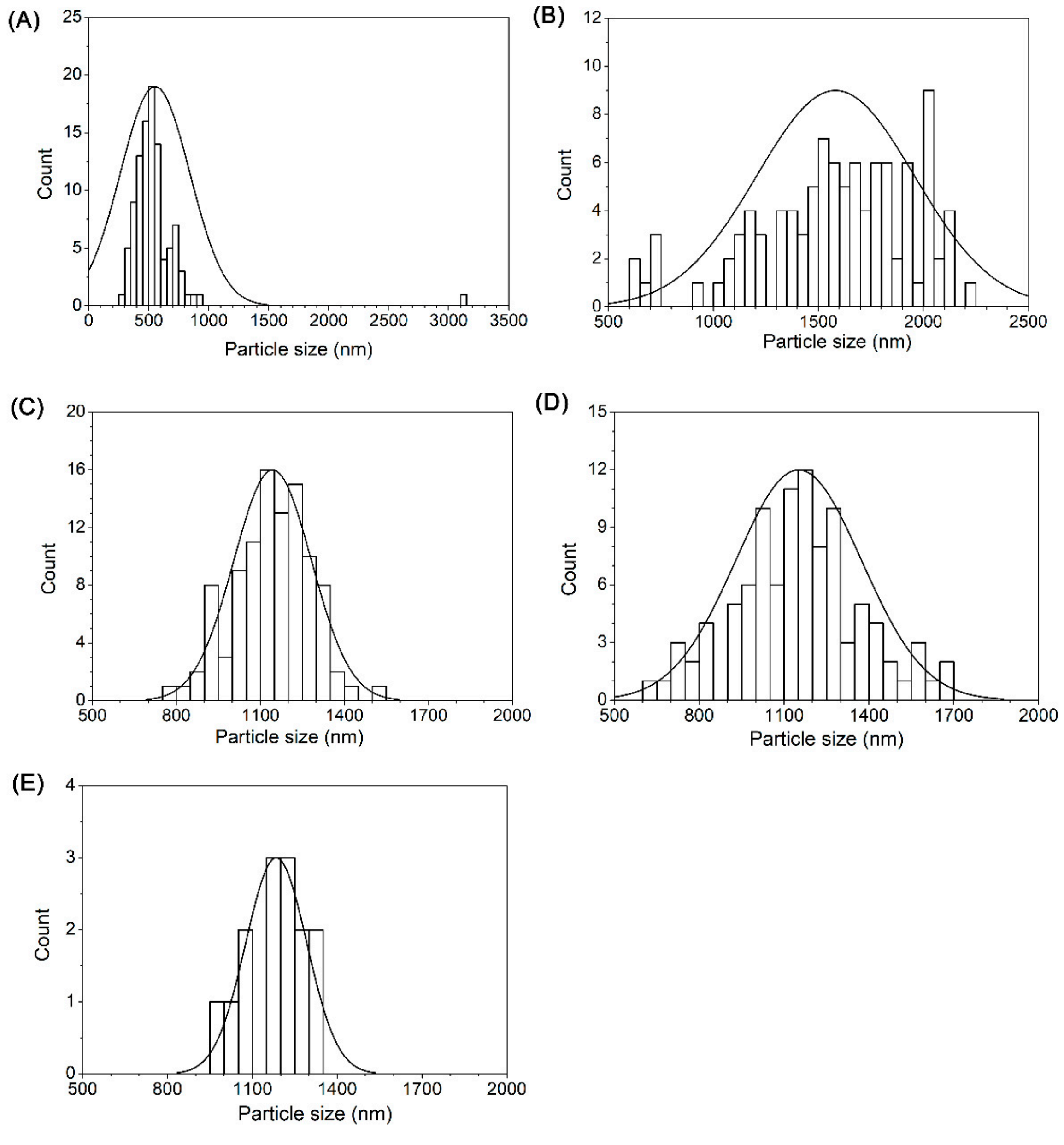

Figure S1 Size distribution of ZSM-5 grains in Figure 2 (A: A_1, B: A_2, C: A_3, D: A_4, E: A_5)

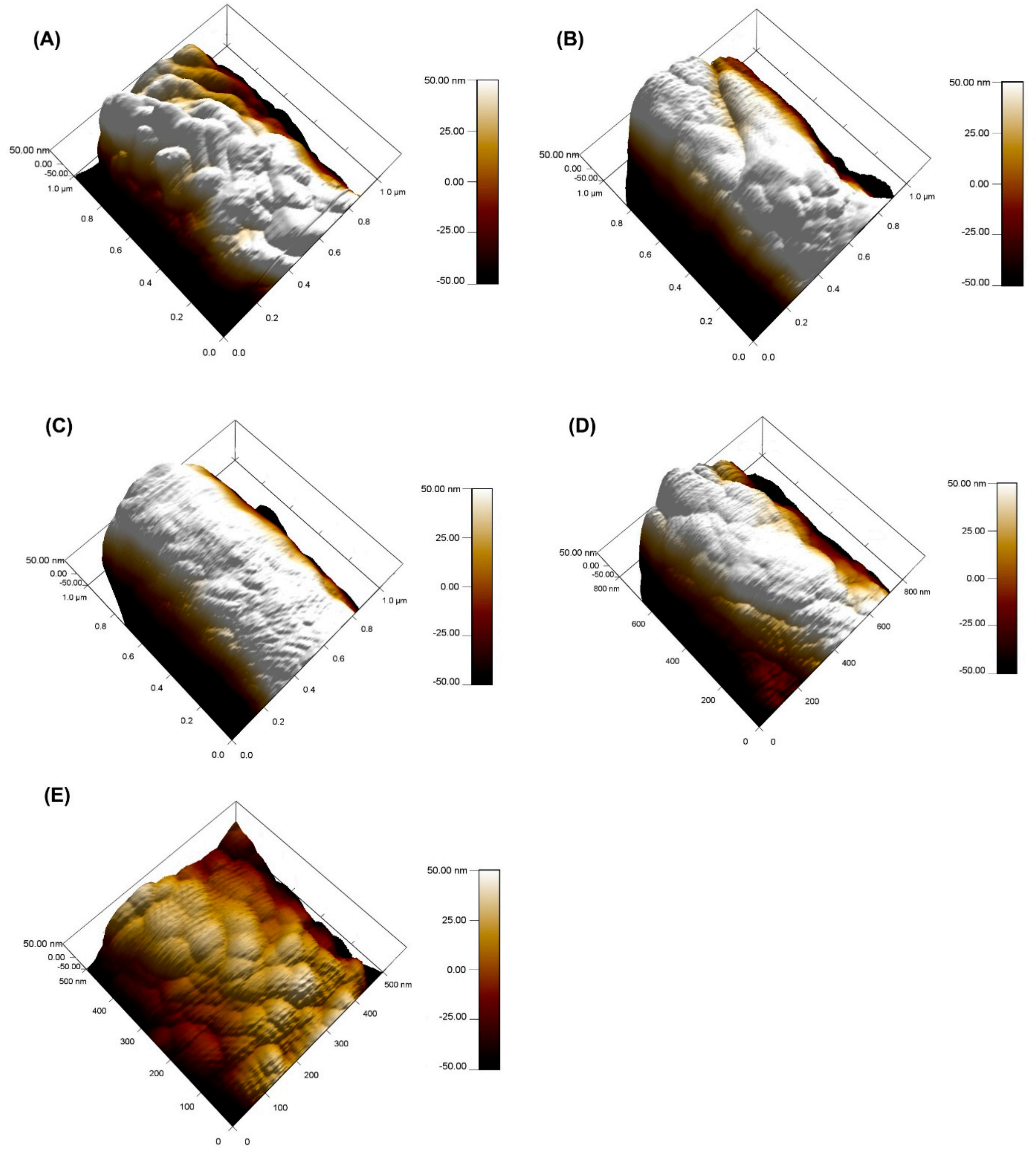

Figure S2 AFM images of ZSM-5 with different SARs. A_1 (A), A_2 (B), A_3 (C), A_4 (D), A_5 (E).

Table S1 Roughness of ZSM-5 with different SARs

Sample	R_q (nm)	R_q (nm)	R_q (nm)	Average (nm)	STD	R_a (nm)	R_a (nm)	R_a (nm)	Average (nm)	STD
A_1	73.420	73.129	72.525	73.02	0.46	60.083	59.12	58.157	59.12	0.96
A_2	68.348	67.839	66.336	67.51	1.05	52.313	53.312	54.311	53.31	1.00
A_3	55.611	55.023	56.725	55.79	0.86	45.799	44.332	42.865	44.33	1.47
A_4	57.623	55.937	56.423	56.66	0.87	43.153	42.862	42.571	42.86	0.29
A_5	27.402	28.203	27.54	27.72	0.43	21.452	22.135	22.818	22.14	0.68

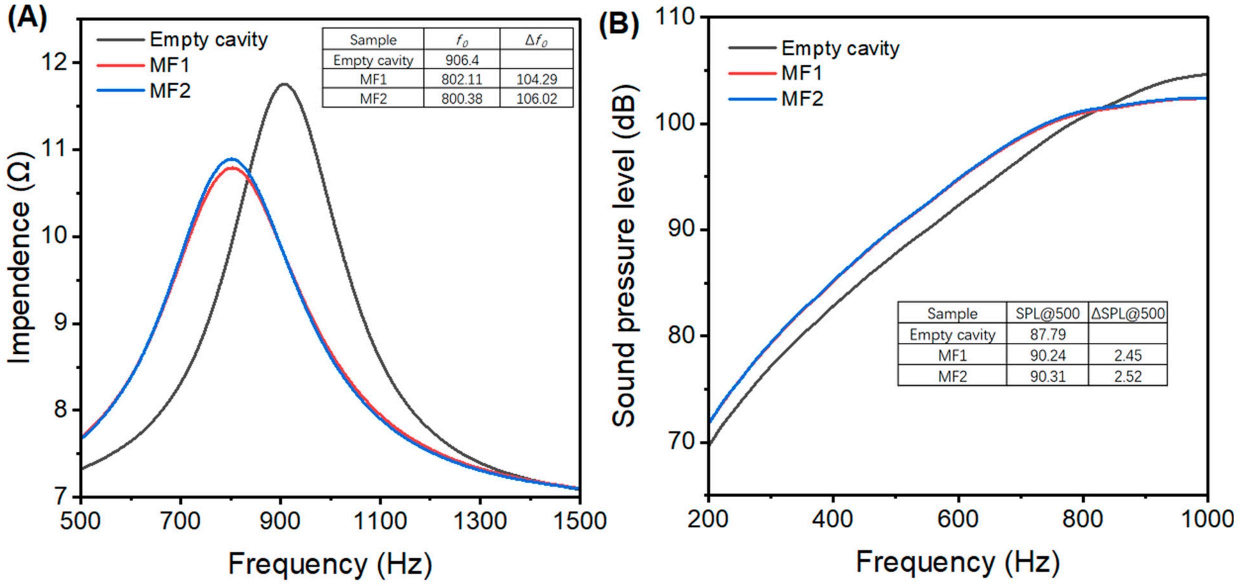

Figure S3 Impendence curve (A) and frequency response curve (B) of sound-absorbing cotton.

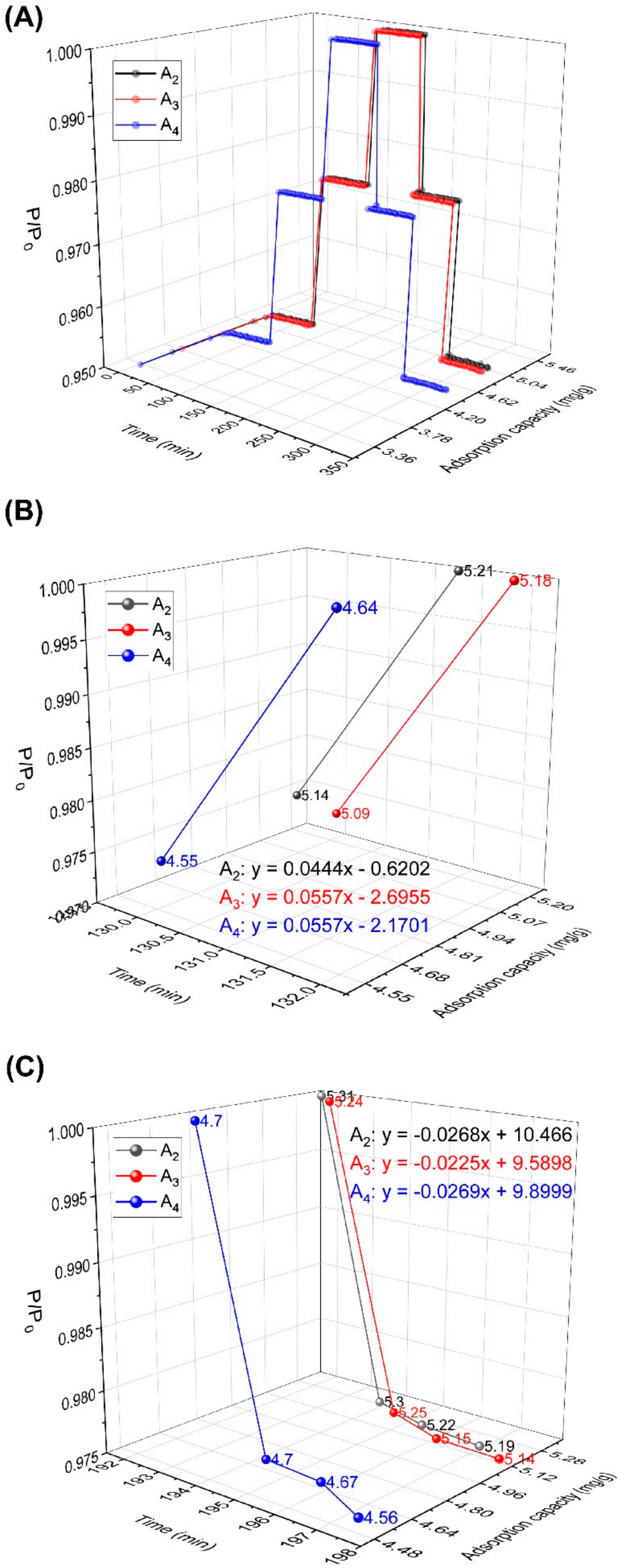

Figure S4 Adsorption capacity-Time curve (A), adsorption rate when P/P_0 increase from 0.975 to 1.0 (B), desorption rate when P/P_0 decrease from 1.0 to 0.975 (C).

Table S2 Detailed elemental compositions of ZSM-5 zeolite samples with different SARs.

Sample	Na2O (wt%)	Al2O3 (wt%)	SiO2 (wt%)	P2O5 (wt%)	SO3 (wt%)	Cl (wt%)	K2O (wt%)	Fe2O3 (wt%)	ZnO (wt%)	ZrO2 (wt%)
A_1	1.53	1.88	96.29	0.19	0.05	0.01	0.01	0.01	0.01	0.02
A_2	0.64	0.97	98.10	0.21	0.05	0.00	0.00	0.00	0.01	0.02
A_3	0.22	0.28	99.31	0.11	0.05	0.01	0.00	0.01	0.00	0.02
A_4	0.20	0.14	99.35	0.17	0.06	0.01	0.00	0.00	0.00	0.06
A_5	0.17	0.03	99.59	0.09	0.06	0.00	0.00	0.01	0.01	0.05
